# Accounting for all territorial emissions and sinks is important for development of climate mitigation policies

**DOI:** 10.1186/s13021-021-00173-8

**Published:** 2021-04-10

**Authors:** Anders Lindroth, Lars Tranvik

**Affiliations:** 1 Department of Physical Geography and Ecosystem Science, Lund University, Lund, Sweden; 2 Department of Ecology and Genetics, Uppsala University, Uppsala, Sweden

**Keywords:** Territorial carbon balance, Emissions from inland waters, Sweden’s mitigation policy

## Abstract

The Paris agreement identifies the importance of the conservation, or better, increase of the land carbon sink. In this respect, the mitigation policies of many forest rich countries rely heavily on products from forests as well as on the land sink. Here we demonstrate that Sweden’s land sink, which is critical in order to achieve zero net emissions by 2045 and negative emissions thereafter, is reduced to less than half when accounting for emissions from wetlands, lakes and running waters. This should have implications for the development of Sweden’s mitigation policy. National as well as the emerging global inventory of sources and sinks need to consider the entire territory to allow accurate guidance of future mitigation of climate change.

## Background

The Paris agreement on climate change [1] has led to sharpening of emission reduction targets in many countries. The emission pathways leading to a global warming not higher than 1.5 ºC in 2100 are based on the state of the global carbon budget, climate sensitivity, lag times and different emission scenarios [2]. In order to reach the 1.5 degree target the atmospheric CO_2_ concentration in 2100 must remain below the 2016 level [2] which at that time was approximately 400 ppm [3]. The calculation of the pathways is based on the net emissions into the atmosphere. Hence, all emissions and sinks, both anthropogenic and natural, count. Still, the integrated overall net emissions are seldom discussed in the context of emission targets. Most focus so far has been on reduction of fossil fuel emissions and partly also on reduction of emissions from deforestation.

The global carbon budget is a closed system and since we know the state of the atmosphere quite precisely, we can calculate how the mean concentration of CO_2_ eqivalents (CO_2eq_) in the atmosphere changes depending on how much CO_2_ and other greenhouse gases are injected or removed in/out of the atmosphere, and thus, based on the climate’s sensitivity to CO_2_, CH4 and other gases also the impact on the temperature of the atmosphere. But the situation is different at the national level. National carbon budgets are not closed systems. All fluxes, lateral and vertical, crossing a nations borders must be known in order to estimate the impact on the atmospheric CO_2eq_ concentration by a particular nation.

## Main text

So, how is this important for the climate mitigation work? We will illustrate this with the case of Sweden. Sweden has adopted very ambitious emission reduction goals [4]: “by 2045, Sweden will have net zero emissions of greenhouse gases into the atmosphere and should thereafter achieve negative emissions”. It is also stated that the “net zero emissions” should be achieved with only limited accounting of the Land Use, Land-Use Change, and Forestry (LULUCF) sector but also that this sector can be used for obtaining the negative emissions. Taking this literally, it implies that Sweden has the goal of actually starting to reduce the CO_2_ concentration in the atmosphere by 2045. In the latest national emission report to the UNFCC by Sweden for year 2018 [5], the emissions amounted to 51.8 M ton of CO_2_eqivalents (CO_2eq_) and the sink in the LULUCF sector was 42.0 M ton of CO_2eq_. The reduction in emissions relative to 1990 was 27% which means about 1% per year. In order to achieve net zero emissions by 2045 the decrease in the rate of emissions must therefore be much higher.

The emission reductions have to a large extent been obtained in the energy sector by utilizing more products from forests to replace fossil fuels. This has so far been possible without reduction of the sink in the LULUCF sector. However, the demand for bioenergy is now heavily increasing, and practically all initiatives are pointing at the forest as the main resource. The average harvest level including other losses has been about 80% of the annual growth in production forests [6] during the last decades, and only a minor fraction, ca. 20% of the harvest is transformed into long-term carbon storage. An increasing demand will most likely increase the harvested fraction of the annual growth and thus also reduce the forest carbon sink [7]. Most of today’s forest sink is in the living biomass with only a smaller fraction, ca 25% going into the soil pool. Considering the time-lag from harvest to re-establishment of a forest carbon sink, increased use of forest products is contrary to the ambition of achieving negative emissions after 2045.

An important question in this context is how well the National Inventory Reports (NIR) to the United Nations Framework Convention on Climate Change [1] represent the emissions and sinks of the territory of Sweden. As stated above, this is crucial when to judge the total climate impact of a country. We don’t discuss the emission inventories from industry, transport, livestock enteric fermentation, etc. or the lateral import/export of carbon but will focus on the LULUCF sector, as it is defined in the National Inventory Report. The methods used to calculate emissions and removals are in accordance with the IPCC 2006 Guidelines for National Greenhouse Gas Inventories, IPCC supplementary guidelines for Kyoto Protocol LULUCF, and the 2013 Supplement to the 2006 IPCC Guidelines for National Greenhouse Gas Inventories. The inventory seems to be quite complete since [5] “All land areas are inventoried in the field except high mountains, military impediments and urban land. We believe that their relative importance for the Swedish GHG inventory is small. The inventory of the LULUCF-sector is complete in the sense that all carbon pools and other sources, where methods are provided in the 2006 GL, are reported for land use categories that are considered managed.” A key word here is “managed”. According to the guidelines the inventory should only consider managed land but according to those responsible for producing the report, all productive forests including set aside forests are included. Thus, all forest land that has any significant impact on the sink should be included.

So, what is missing? Within the territory of Sweden we also have mires, lakes and rivers that have processes involving exchanges of greenhouse gases. None of these categories are included in the Swedish NIR reporting. Mires are typically sinks of carbon dioxide and sources of methane and both the uptake of CO_2_ and the emission of CH_4_ depend strongly on vegetation type [8] and it is therefore not so easy to estimate the net emission expressed as CO_2eq_ for the whole mire area of Sweden. Moreover, their greenhouse gas balance is sensitive to drainage and rewetting. However, one such attempt was made [9] and the conclusion was that mires (including bogs, fens, and marshes) in Sweden are emitting ca. 2.5 M ton CO_2eq_ annually. Lakes, streams and rivers emit substantial amounts of carbon originating from the terrestrial environment. Based on data from [10] we estimate emission of CO_2_ from lakes in Sweden to 8.25 M ton CO_2_ and [11] estimated the corresponding value for rivers and streams to 9.9 M ton CO_2_. In addition, lakes [12] and streams [11] emit methane, additionally corresponding to 2.2 and 0.5 M ton CO_2eq_, respectively. This makes a total net emission of 23.4 M ton CO_2eq_ for mires and inland waters. Adding this to the reported LULUCF sink, we estimate the territorial land area sink in Sweden to ca 18.6 M ton CO2eq. Hence, when considering mires, lakes and running waters, the national sink is reduced to less than half of that reported in the NIR. In 2019, The IPCC has published a refinement to the Guidelines for National Greenhouse Gas Inventories [13] which includes areas that are flooded due to human activities. This is likely to result in only marginal improvement of future National Inventory Reports, since most wetlands and inland waters are still excluded.

## Conclusions

Territorial emissions of atmospheric CO_2eq_ are the result of numerous sources and sinks. A full account of those is crucial to guide mitigation of climate change. Here, we show that in the case of Sweden, a complete inventory reveals a weaker territorial C sink than what is currently considered, i.e. the complete land sink balances less of the national emissions than is offset by the reported LULUCF sink. Although there is considerable uncertainty in these numbers as well as potentially unaccounted sources and sinks, they demonstrate that care should be taken to preserve the forest carbon sink, rather than intensifying harvesting of forest biomass. Without high ambitions in maintaining carbon sinks, the Swedish goal of obtaining negative emissions after 2045 cannot be retained. We recommend that guidelines and methods for estimation of total territorial greenhouse gas balances should be developed to make such estimations possible in all countries. Moreover, complete territorial inventories and their certainty are seriously hampered by lack of fundamental data. Improved data collection and complete territorial inventories are important for policy development of climate mitigation strategies.

## Data Availability

All data comes from published sources.
